# Ethyl 6-methyl-2-oxo-4-[4-(1*H*-tetra­zol-5-yl)phen­yl]-1,2,3,4-tetra­hydro­pyrimidine-5-carboxyl­ate–di­methyl­formamide–water (2/1/1)

**DOI:** 10.1107/S1600536813032224

**Published:** 2013-12-04

**Authors:** Hua-Yong Ouyang, Yi-Qi Chang, Lu Zhao

**Affiliations:** a Department of Chemical Engineering, Nanjing College of Chemical Technology, Nanjing 210048, People’s Republic of China; b Department of Applied Chemistry, Nanjing College of Chemical Technology, Nanjing 210048, People’s Republic of China

## Abstract

The asymmetric unit of the title compound, 2C_15_H_16_N_6_O_3_·C_3_H_7_NO·H_2_O, contains two independent ethyl 6-methyl-2-oxo-4-[4-(1*H*-tetra­zol-5-yl)phen­yl]-1,2,3,4-tetra­hydro­pyrim­id­ine-5-carboxyl­ate mol­ecules, in which the dihedral angles between the tetra­zole and benzene rings are 20.54 (12) and 12.13 (12)°. An intra­molecular C—H⋯O hydrogen bond occurs in each mol­ecule. In the crystal, N—H⋯O, N—H⋯N, O—H⋯O and O—H⋯N hydrogen bonds, as well as weak C—H⋯O and C—H⋯N hydrogen bonds, link the mol­ecules into a three-dimensional supra­molecular architecture. π–π stacking is also observed between parallel tetra­zole rings of adjacent mol­ecules, the centroid–centroid distance being 3.482 (6) Å.

## Related literature   

For applications of hydro­pyrimidine derivatives and related compounds, see: Atwal *et al.* (1990 ▶); Kappe & Stadler (2004 ▶). 
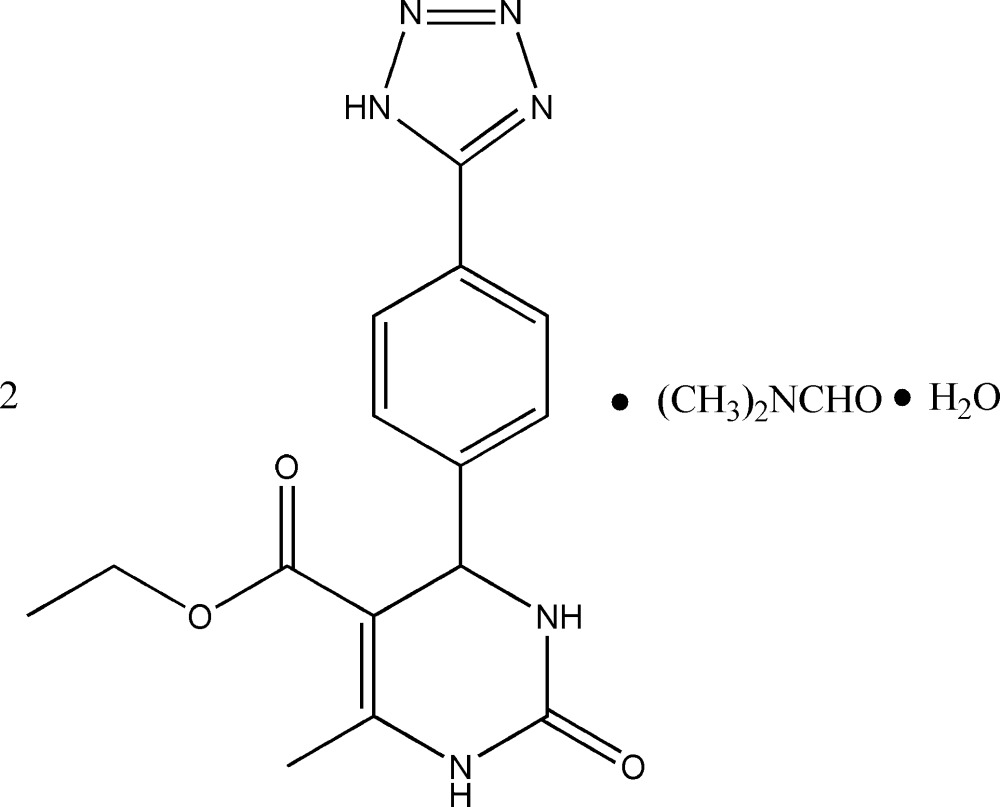


## Experimental   

### 

#### Crystal data  

2C_15_H_16_N_6_O_3_·C_3_H_7_NO·H_2_O
*M* *_r_* = 747.79
Triclinic, 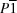

*a* = 10.198 (2) Å
*b* = 13.262 (3) Å
*c* = 13.771 (3) Å
α = 81.14 (3)°
β = 73.32 (3)°
γ = 81.14 (3)°
*V* = 1750.9 (6) Å^3^
*Z* = 2
Mo *K*α radiation
μ = 0.11 mm^−1^
*T* = 293 K
0.40 × 0.30 × 0.20 mm

#### Data collection  

Rigaku Mercury2 diffractometer
Absorption correction: multi-scan (*CrystalClear*; Rigaku, 2005 ▶) *T*_min_ = 0.832, *T*_max_ = 1.000
18496 measured reflections
7997 independent reflections
5573 reflections with *I* > 2σ(*I*)
*R*_int_ = 0.037

#### Refinement  

*R*[*F* ^2^ > 2σ(*F* ^2^)] = 0.059
*wR*(*F* ^2^) = 0.157
*S* = 1.12
7997 reflections
487 parameters
H-atom parameters constrained
Δρ_max_ = 0.38 e Å^−3^
Δρ_min_ = −0.29 e Å^−3^

### 

Data collection: *CrystalClear* (Rigaku, 2005 ▶); cell refinement: *CrystalClear*; data reduction: *CrystalClear*; program(s) used to solve structure: *SHELXS97* (Sheldrick, 2008 ▶); program(s) used to refine structure: *SHELXL97* (Sheldrick, 2008 ▶); molecular graphics: *SHELXTL* (Sheldrick, 2008 ▶); software used to prepare material for publication: *SHELXTL*.

## Supplementary Material

Crystal structure: contains datablock(s) I, New_Global_Publ_Block. DOI: 10.1107/S1600536813032224/xu5754sup1.cif

Structure factors: contains datablock(s) I. DOI: 10.1107/S1600536813032224/xu5754Isup2.hkl

Supporting information file. DOI: 10.1107/S1600536813032224/xu5754Isup3.cml

Additional supporting information:  crystallographic information; 3D view; checkCIF report

## Figures and Tables

**Table 1:** Hydrogen-bond geometry (Å, °)

*D*—H⋯*A*	*D*—H	H⋯*A*	*D*⋯*A*	*D*—H⋯*A*
N5—H5*A*⋯O1*W*	0.86	1.80	2.646 (3)	170
N9—H9*A*⋯O7	0.86	1.82	2.683 (3)	176
N10—H10*B*⋯O2	0.86	1.98	2.801 (2)	160
N11—H11*B*⋯N2^i^	0.86	2.26	3.014 (3)	147
N12—H12*A*⋯O1	0.86	2.04	2.884 (2)	165
N13—H13*B*⋯O7^ii^	0.86	2.44	3.176 (3)	144
O1*W*—H1*WA*⋯N8^ii^	0.85	2.17	2.985 (3)	160
O1*W*—H1*WB*⋯O1^iii^	0.85	2.01	2.677 (2)	135
C5—H5*D*⋯O3	0.96	2.07	2.817 (3)	133
C16—H16*A*⋯O5	0.96	2.03	2.781 (3)	133
C26—H26*A*⋯O7	0.93	2.59	3.431 (3)	151
C32—H32*A*⋯N3^iv^	0.96	2.53	3.463 (4)	164
C33—H33*A*⋯N3^iv^	1.00	2.53	3.506 (3)	164

Symmetry codes: (i) 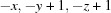; (ii) 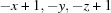; (iii) 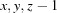; (iv) 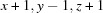.

**Fig. 1.**
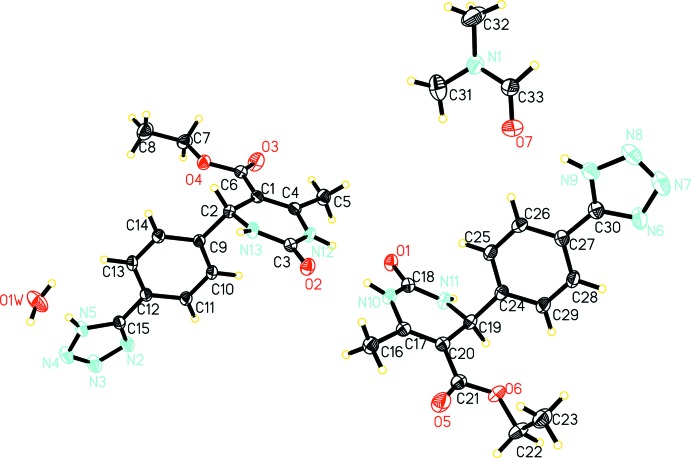
Perspective structure of the title compound, showing the atom-numbering scheme.

**Fig. 2.**
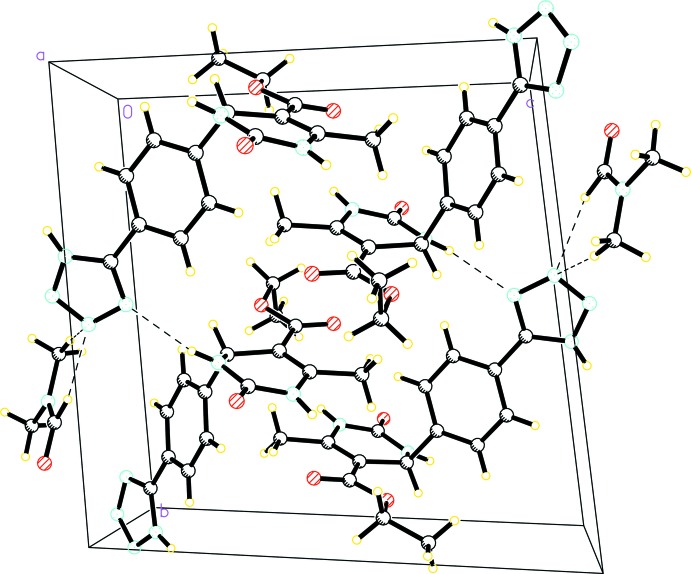
The crystal packing of the title compound viewed along the a axis showing the hydrogen bondings network.

## supplementary crystallographic information

### 1. Comment

3,4-Dihydropyimidin-2(1H)-ones have shown good drug activity (Atwal *et al.*, 1990; Kappe & Stadler, 2004). The tetrazoles have been showed that analogs of biologically active carboxylic acids in which the carboxyl group is replaced by a 5-tetrazolyl group might interfere with the normal utilization of the respective carboxylic acids. The Biginelli derivative was obtained from p-cyanobenzaldehyde, that was used to yield tetrazole derivative. Here we report the synthesis and crystal structure of the title compound (Fig. 1).

The bond distances and bond angles in the title compound agree very well with the corresponding distances and angles reported for a closely related compound. There are two biginelli derivatives and two solvate molecules in an asymmetric unit. The inter-molecular N–H···O and C–H···O hydrogen bonds link the compound to two-dimensional structure (Table 1), in which they may be effective in the stabilization of the structure. π-π contact between the tetrazole rings, Cg1-Cg1^i^ [symmetry code: (i) -x, 1-y, -z, where Cg1 and Cg1^i^ are centroids of the rings (N2-C15)] may further stabilize the structure, centroid-centroid distance of 3.482 (6) Å.

### 2. Experimental

Cyanobenzaldehyd and ethyl acetoacetate and urea (1:1:1) was added to round-bottom flask without solvent under nitrogen. The temperature was raised to 80°C in one hour gradually and the mixture was stirred at this temperature for 12 h. The system was treated with 30 ml of ethanol 95% and cooled. The precipitate was filtered and washed with a small amount of ethanol 95%. The above-mentioned compound (10 mmol) was added to sodium azide (15 mmol) and ammonia chloride (12 mmol) with DMF solvent. The temperature was raised to 115°C in one hour gradually and the mixture was stirred at this temperature for 36 h. The system was treated with 30 ml of water and cooled. The precipitate was filtered at pH 3. Single crystals suitable for X-ray diffraction analysis were obtained from slow evaporation of a solution of the title compound in DMF/water at room temperature

### 3. Refinement

H-atoms bonded to the C-atoms were positioned geometrically and refined using a riding model with C—H = 0.93–1.00 Å and *U*_iso_(H) = 1.5*U*_eq_(C) for methyl H atoms and 1.2U_eq_(C) for the others. H-atoms bonded to the N-atoms and O-atom were located from a difference Fourier map and refined in riding mode with O—H = 0.85 and N—H 0.86 Å, U_iso_(H) = 1.5U_eq_(O) and 1.2U_eq_(N).

### Crystal data

**Table:**

2C_15_H_16_N_6_O_3_·C_3_H_7_NO·H_2_O	*Z* = 2
*M**_r_* = 747.79	*F*(000) = 788
Triclinic, *P*1	*D*_x_ = 1.418 Mg m^−^^3^
Hall symbol: -P 1	Mo *K*α radiation, λ = 0.71073 Å
*a* = 10.198 (2) Å	Cell parameters from 7997 reflections
*b* = 13.262 (3) Å	θ = 2.6–27.5°
*c* = 13.771 (3) Å	µ = 0.11 mm^−^^1^
α = 81.14 (3)°	*T* = 293 K
β = 73.32 (3)°	Prism, colorless
γ = 81.14 (3)°	0.40 × 0.30 × 0.20 mm
*V* = 1750.9 (6) Å^3^	

### Data collection

**Table:**

Rigaku Mercury2 diffractometer	7997 independent reflections
Radiation source: fine-focus sealed tube	5573 reflections with *I* > 2σ(*I*)
Graphite monochromator	*R*_int_ = 0.037
Detector resolution: 13.6612 pixels mm^-1^	θ_max_ = 27.5°, θ_min_ = 3.1°
CCD_Profile_fitting scans	*h* = −13→13
Absorption correction: multi-scan (*CrystalClear*; Rigaku, 2005)	*k* = −17→17
*T*_min_ = 0.832, *T*_max_ = 1.000	*l* = −17→17
18496 measured reflections	

### Refinement

**Table:**

Refinement on *F*^2^	Primary atom site location: structure-invariant direct methods
Least-squares matrix: full	Secondary atom site location: difference Fourier map
*R*[*F*^2^ > 2σ(*F*^2^)] = 0.059	Hydrogen site location: inferred from neighbouring sites
*wR*(*F*^2^) = 0.157	H-atom parameters constrained
*S* = 1.12	*w* = 1/[σ^2^(*F*_o_^2^) + (0.0633*P*)^2^ + 0.4646*P*] where *P* = (*F*_o_^2^ + 2*F*_c_^2^)/3
7997 reflections	(Δ/σ)_max_ = 0.011
487 parameters	Δρ_max_ = 0.38 e Å^−^^3^
0 restraints	Δρ_min_ = −0.29 e Å^−^^3^

### Special details

**Table:**

Geometry. All e.s.d.'s (except the e.s.d. in the dihedral angle between two l.s. planes) are estimated using the full covariance matrix. The cell e.s.d.'s are taken into account individually in the estimation of e.s.d.'s in distances, angles and torsion angles; correlations between e.s.d.'s in cell parameters are only used when they are defined by crystal symmetry. An approximate (isotropic) treatment of cell e.s.d.'s is used for estimating e.s.d.'s involving l.s. planes.
Refinement. Refinement of *F*^2^ against ALL reflections. The weighted *R*-factor *wR* and goodness of fit *S* are based on *F*^2^, conventional *R*-factors *R* are based on *F*, with *F* set to zero for negative *F*^2^. The threshold expression of *F*^2^ > σ(*F*^2^) is used only for calculating *R*-factors(gt) *etc*. and is not relevant to the choice of reflections for refinement. *R*-factors based on *F*^2^ are statistically about twice as large as those based on *F*, and *R*- factors based on ALL data will be even larger.

### Fractional atomic coordinates and isotropic or equivalent isotropic displacement parameters (Å^2^)

**Table:**

	*x*	*y*	*z*	*U*_iso_*/*U*_eq_	
C1	−0.0641 (2)	0.12944 (14)	0.48373 (14)	0.0307 (4)	
C2	0.0311 (2)	0.12423 (15)	0.37903 (14)	0.0307 (4)	
H2A	0.0349	0.0553	0.3602	0.037*	
C3	0.1996 (2)	0.18600 (15)	0.44688 (15)	0.0328 (4)	
C4	−0.0284 (2)	0.16860 (15)	0.55451 (15)	0.0323 (4)	
C6	−0.1986 (2)	0.09564 (15)	0.50272 (16)	0.0347 (4)	
C7	−0.3396 (2)	0.02727 (19)	0.42842 (19)	0.0472 (6)	
H7A	−0.4063	0.0881	0.4305	0.057*	
H7B	−0.3708	−0.0194	0.4896	0.057*	
C8	−0.3256 (3)	−0.0229 (2)	0.3366 (2)	0.0636 (7)	
H8A	−0.4132	−0.0422	0.3382	0.095*	
H8B	−0.2597	−0.0831	0.3355	0.095*	
H8C	−0.2948	0.0240	0.2766	0.095*	
C9	−0.01594 (19)	0.20105 (15)	0.30001 (14)	0.0293 (4)	
C10	−0.0476 (2)	0.30276 (16)	0.31468 (15)	0.0374 (5)	
H10A	−0.0421	0.3237	0.3747	0.045*	
C11	−0.0868 (2)	0.37322 (16)	0.24300 (15)	0.0380 (5)	
H11A	−0.1084	0.4419	0.2543	0.046*	
C12	−0.0948 (2)	0.34373 (15)	0.15389 (14)	0.0317 (4)	
C13	−0.0661 (2)	0.24207 (16)	0.13965 (15)	0.0397 (5)	
H13A	−0.0735	0.2209	0.0803	0.048*	
C14	−0.0268 (2)	0.17167 (16)	0.21199 (15)	0.0381 (5)	
H14A	−0.0071	0.1027	0.2014	0.046*	
C15	−0.1364 (2)	0.41983 (16)	0.07781 (15)	0.0331 (4)	
C16	0.5826 (2)	0.33833 (18)	0.43776 (16)	0.0440 (5)	
H16A	0.6658	0.3705	0.4199	0.066*	
H16B	0.5233	0.3729	0.3970	0.066*	
H16C	0.6048	0.2676	0.4256	0.066*	
C17	0.5117 (2)	0.34458 (15)	0.54674 (15)	0.0330 (4)	
C18	0.2936 (2)	0.31894 (15)	0.66511 (15)	0.0336 (4)	
C19	0.4688 (2)	0.38080 (16)	0.72315 (15)	0.0339 (4)	
H19A	0.4668	0.4463	0.7485	0.041*	
C20	0.5511 (2)	0.38809 (15)	0.61304 (15)	0.0337 (4)	
C21	0.6726 (2)	0.44166 (16)	0.58067 (18)	0.0396 (5)	
C22	0.8083 (2)	0.53775 (19)	0.6345 (2)	0.0538 (6)	
H22A	0.7941	0.5882	0.6817	0.065*	
H22B	0.8268	0.5735	0.5659	0.065*	
C23	0.9280 (3)	0.4622 (2)	0.6440 (2)	0.0610 (7)	
H23A	1.0084	0.4971	0.6298	0.091*	
H23B	0.9432	0.4131	0.5964	0.091*	
H23C	0.9102	0.4273	0.7122	0.091*	
C24	0.5315 (2)	0.29673 (15)	0.78896 (15)	0.0327 (4)	
C25	0.5135 (2)	0.19572 (17)	0.79301 (18)	0.0419 (5)	
H25A	0.4583	0.1785	0.7563	0.050*	
C26	0.5750 (2)	0.12007 (17)	0.84981 (17)	0.0426 (5)	
H26A	0.5612	0.0520	0.8515	0.051*	
C27	0.6568 (2)	0.14369 (16)	0.90431 (15)	0.0357 (5)	
C28	0.6745 (2)	0.24440 (18)	0.90086 (17)	0.0445 (5)	
H28A	0.7301	0.2617	0.9373	0.053*	
C29	0.6123 (2)	0.31926 (17)	0.84517 (17)	0.0431 (5)	
H29A	0.6246	0.3874	0.8450	0.052*	
C30	0.7271 (2)	0.06657 (17)	0.96436 (16)	0.0402 (5)	
C31	0.4547 (3)	−0.2117 (3)	0.7892 (2)	0.0740 (9)	
H31A	0.4796	−0.1429	0.7727	0.111*	
H31B	0.3594	−0.2101	0.8269	0.111*	
H31C	0.4691	−0.2420	0.7274	0.111*	
C32	0.5170 (4)	−0.3773 (2)	0.8813 (3)	0.0850 (10)	
H32A	0.5786	−0.4086	0.9214	0.127*	
H32B	0.5345	−0.4125	0.8222	0.127*	
H32C	0.4235	−0.3816	0.9214	0.127*	
C33	0.6262 (3)	−0.22938 (19)	0.8775 (2)	0.0549 (6)	
H33A	0.6724	−0.2820	0.9215	0.066*	
N1	0.5385 (2)	−0.27157 (15)	0.84984 (16)	0.0497 (5)	
N2	−0.2044 (2)	0.51006 (14)	0.09321 (14)	0.0452 (5)	
N3	−0.2227 (2)	0.55284 (16)	0.00233 (16)	0.0542 (5)	
N4	−0.1683 (2)	0.49185 (16)	−0.06570 (15)	0.0516 (5)	
N5	−0.11397 (19)	0.40810 (14)	−0.01895 (13)	0.0401 (4)	
H5A	−0.0709	0.3546	−0.0473	0.048*	
N6	0.7876 (2)	0.08564 (17)	1.03020 (16)	0.0591 (6)	
N7	0.8391 (3)	−0.0052 (2)	1.06887 (18)	0.0707 (7)	
N8	0.8126 (2)	−0.07778 (18)	1.02923 (17)	0.0621 (6)	
N9	0.7422 (2)	−0.03366 (15)	0.96288 (15)	0.0500 (5)	
H9A	0.7116	−0.0654	0.9254	0.060*	
N10	0.39147 (17)	0.30075 (13)	0.57831 (13)	0.0363 (4)	
H10B	0.3776	0.2594	0.5408	0.044*	
N11	0.32924 (17)	0.36679 (13)	0.72897 (13)	0.0364 (4)	
H11B	0.2645	0.3915	0.7777	0.044*	
N12	0.09736 (17)	0.20394 (13)	0.53140 (12)	0.0354 (4)	
H12A	0.1120	0.2398	0.5734	0.042*	
N13	0.16843 (17)	0.13960 (13)	0.37938 (12)	0.0351 (4)	
H13B	0.2358	0.1165	0.3314	0.042*	
O1	0.17888 (14)	0.29226 (12)	0.68075 (11)	0.0411 (4)	
O2	0.31418 (15)	0.21023 (12)	0.43537 (11)	0.0430 (4)	
O3	−0.29294 (17)	0.10389 (15)	0.57646 (13)	0.0583 (5)	
O4	−0.20729 (15)	0.05470 (12)	0.42274 (11)	0.0425 (4)	
O5	0.75258 (19)	0.44626 (16)	0.49888 (14)	0.0670 (6)	
O6	0.68567 (16)	0.48804 (12)	0.65585 (12)	0.0468 (4)	
O7	0.6507 (2)	−0.14120 (13)	0.85083 (15)	0.0659 (5)	
O1W	0.0354 (2)	0.25890 (15)	−0.12360 (13)	0.0753 (6)	
H1WA	0.0624	0.2107	−0.0829	0.113*	
H1WB	0.1049	0.2824	−0.1668	0.113*	
C5	−0.1111 (2)	0.17917 (19)	0.66052 (16)	0.0464 (6)	
H5D	−0.1977	0.1530	0.6722	0.070*	
H5E	−0.1272	0.2504	0.6713	0.070*	
H5B	−0.0621	0.1410	0.7070	0.070*	

### Atomic displacement parameters (Å^2^)

**Table:**

	*U*^11^	*U*^22^	*U*^33^	*U*^12^	*U*^13^	*U*^23^
C1	0.0338 (11)	0.0293 (10)	0.0280 (10)	−0.0028 (8)	−0.0087 (8)	−0.0010 (8)
C2	0.0338 (10)	0.0317 (10)	0.0286 (10)	−0.0053 (8)	−0.0100 (8)	−0.0049 (8)
C3	0.0350 (11)	0.0341 (10)	0.0299 (10)	−0.0044 (8)	−0.0098 (9)	−0.0028 (8)
C4	0.0360 (11)	0.0308 (10)	0.0291 (10)	−0.0041 (8)	−0.0081 (8)	−0.0017 (8)
C6	0.0390 (12)	0.0329 (10)	0.0327 (10)	−0.0062 (9)	−0.0106 (9)	−0.0013 (8)
C7	0.0404 (13)	0.0482 (13)	0.0601 (15)	−0.0156 (10)	−0.0219 (11)	−0.0013 (11)
C8	0.0693 (18)	0.0600 (17)	0.0775 (19)	−0.0116 (14)	−0.0409 (16)	−0.0117 (14)
C9	0.0290 (10)	0.0326 (10)	0.0273 (9)	−0.0072 (8)	−0.0076 (8)	−0.0030 (8)
C10	0.0505 (13)	0.0365 (11)	0.0304 (10)	−0.0063 (9)	−0.0165 (9)	−0.0077 (9)
C11	0.0514 (13)	0.0313 (10)	0.0338 (11)	−0.0018 (9)	−0.0155 (10)	−0.0071 (9)
C12	0.0309 (10)	0.0371 (11)	0.0279 (9)	−0.0061 (8)	−0.0089 (8)	−0.0023 (8)
C13	0.0544 (14)	0.0416 (12)	0.0271 (10)	−0.0062 (10)	−0.0146 (10)	−0.0088 (9)
C14	0.0530 (13)	0.0311 (10)	0.0321 (10)	−0.0035 (9)	−0.0130 (10)	−0.0074 (9)
C15	0.0329 (11)	0.0385 (11)	0.0285 (10)	−0.0057 (9)	−0.0084 (8)	−0.0034 (8)
C16	0.0414 (13)	0.0534 (14)	0.0369 (11)	−0.0098 (10)	−0.0048 (10)	−0.0113 (10)
C17	0.0319 (11)	0.0304 (10)	0.0361 (11)	−0.0013 (8)	−0.0084 (9)	−0.0062 (8)
C18	0.0306 (11)	0.0362 (11)	0.0333 (10)	−0.0001 (8)	−0.0088 (9)	−0.0057 (9)
C19	0.0331 (11)	0.0343 (10)	0.0377 (11)	−0.0041 (8)	−0.0109 (9)	−0.0118 (9)
C20	0.0324 (11)	0.0322 (10)	0.0364 (11)	−0.0027 (8)	−0.0093 (9)	−0.0050 (8)
C21	0.0384 (12)	0.0371 (11)	0.0455 (13)	−0.0070 (9)	−0.0132 (11)	−0.0055 (10)
C22	0.0470 (14)	0.0442 (13)	0.0767 (18)	−0.0180 (11)	−0.0163 (13)	−0.0141 (12)
C23	0.0523 (16)	0.0581 (16)	0.082 (2)	−0.0135 (12)	−0.0277 (14)	−0.0097 (14)
C24	0.0299 (10)	0.0363 (11)	0.0329 (10)	−0.0028 (8)	−0.0065 (8)	−0.0117 (9)
C25	0.0459 (13)	0.0396 (12)	0.0494 (13)	−0.0081 (10)	−0.0242 (11)	−0.0082 (10)
C26	0.0500 (14)	0.0337 (11)	0.0491 (13)	−0.0099 (10)	−0.0176 (11)	−0.0068 (10)
C27	0.0364 (11)	0.0395 (11)	0.0293 (10)	−0.0007 (9)	−0.0056 (9)	−0.0086 (9)
C28	0.0532 (14)	0.0455 (13)	0.0456 (13)	−0.0081 (10)	−0.0259 (11)	−0.0113 (10)
C29	0.0547 (14)	0.0360 (11)	0.0465 (13)	−0.0070 (10)	−0.0218 (11)	−0.0113 (10)
C30	0.0415 (12)	0.0450 (13)	0.0320 (11)	−0.0004 (10)	−0.0062 (9)	−0.0097 (9)
C31	0.0593 (18)	0.107 (3)	0.0577 (17)	−0.0001 (17)	−0.0247 (15)	−0.0083 (17)
C32	0.086 (2)	0.0555 (18)	0.129 (3)	−0.0184 (16)	−0.043 (2)	−0.0201 (19)
C33	0.0670 (17)	0.0409 (14)	0.0644 (16)	−0.0042 (12)	−0.0321 (14)	−0.0041 (12)
N1	0.0499 (12)	0.0497 (12)	0.0537 (12)	−0.0019 (9)	−0.0186 (10)	−0.0142 (10)
N2	0.0537 (12)	0.0418 (11)	0.0399 (10)	0.0044 (9)	−0.0178 (9)	−0.0036 (8)
N3	0.0645 (14)	0.0504 (12)	0.0478 (12)	0.0087 (10)	−0.0249 (11)	−0.0027 (10)
N4	0.0600 (13)	0.0545 (13)	0.0389 (11)	0.0022 (10)	−0.0207 (10)	0.0042 (10)
N5	0.0455 (11)	0.0437 (10)	0.0306 (9)	−0.0004 (8)	−0.0130 (8)	−0.0023 (8)
N6	0.0727 (15)	0.0600 (14)	0.0522 (12)	0.0076 (11)	−0.0332 (12)	−0.0136 (11)
N7	0.0852 (18)	0.0739 (17)	0.0538 (14)	0.0153 (13)	−0.0335 (13)	−0.0061 (12)
N8	0.0703 (16)	0.0570 (14)	0.0528 (13)	0.0094 (11)	−0.0209 (12)	0.0040 (11)
N9	0.0605 (13)	0.0434 (11)	0.0453 (11)	−0.0013 (9)	−0.0166 (10)	−0.0034 (9)
N10	0.0334 (9)	0.0421 (10)	0.0353 (9)	−0.0072 (7)	−0.0057 (8)	−0.0140 (8)
N11	0.0273 (9)	0.0464 (10)	0.0361 (9)	0.0010 (7)	−0.0062 (7)	−0.0162 (8)
N12	0.0353 (9)	0.0434 (10)	0.0307 (9)	−0.0097 (8)	−0.0076 (7)	−0.0114 (8)
N13	0.0295 (9)	0.0462 (10)	0.0298 (8)	−0.0010 (7)	−0.0070 (7)	−0.0107 (8)
O1	0.0282 (8)	0.0599 (10)	0.0366 (8)	−0.0082 (7)	−0.0067 (6)	−0.0106 (7)
O2	0.0329 (8)	0.0580 (10)	0.0408 (8)	−0.0118 (7)	−0.0081 (7)	−0.0110 (7)
O3	0.0405 (10)	0.0812 (13)	0.0524 (10)	−0.0202 (9)	0.0033 (8)	−0.0228 (9)
O4	0.0410 (9)	0.0513 (9)	0.0401 (8)	−0.0168 (7)	−0.0134 (7)	−0.0043 (7)
O5	0.0564 (12)	0.0963 (15)	0.0508 (11)	−0.0395 (11)	0.0006 (9)	−0.0144 (10)
O6	0.0430 (9)	0.0453 (9)	0.0578 (10)	−0.0151 (7)	−0.0123 (8)	−0.0157 (8)
O7	0.0874 (14)	0.0418 (10)	0.0728 (13)	−0.0136 (9)	−0.0276 (11)	−0.0024 (9)
O1W	0.0929 (15)	0.0643 (12)	0.0405 (10)	0.0199 (11)	0.0065 (10)	0.0026 (9)
C5	0.0467 (13)	0.0597 (15)	0.0323 (11)	−0.0137 (11)	−0.0028 (10)	−0.0115 (10)

### Geometric parameters (Å, º)

**Table:**

C1—C4	1.331 (3)	C22—O6	1.438 (3)
C1—C6	1.449 (3)	C22—C23	1.479 (4)
C1—C2	1.494 (3)	C22—H22A	0.9700
C2—N13	1.448 (2)	C22—H22B	0.9700
C2—C9	1.503 (3)	C23—H23A	0.9600
C2—H2A	0.9800	C23—H23B	0.9600
C3—O2	1.219 (2)	C23—H23C	0.9600
C3—N13	1.323 (3)	C24—C25	1.370 (3)
C3—N12	1.346 (3)	C24—C29	1.371 (3)
C4—N12	1.367 (3)	C25—C26	1.363 (3)
C4—C5	1.477 (3)	C25—H25A	0.9300
C6—O3	1.187 (3)	C26—C27	1.368 (3)
C6—O4	1.331 (2)	C26—H26A	0.9300
C7—O4	1.428 (3)	C27—C28	1.367 (3)
C7—C8	1.480 (4)	C27—C30	1.448 (3)
C7—H7A	0.9700	C28—C29	1.352 (3)
C7—H7B	0.9700	C28—H28A	0.9300
C8—H8A	0.9600	C29—H29A	0.9300
C8—H8B	0.9600	C30—N6	1.306 (3)
C8—H8C	0.9600	C30—N9	1.317 (3)
C9—C14	1.367 (3)	C31—N1	1.433 (3)
C9—C10	1.370 (3)	C31—H31A	0.9600
C10—C11	1.355 (3)	C31—H31B	0.9600
C10—H10A	0.9300	C31—H31C	0.9600
C11—C12	1.371 (3)	C32—N1	1.433 (4)
C11—H11A	0.9300	C32—H32A	0.9600
C12—C13	1.367 (3)	C32—H32B	0.9600
C12—C15	1.447 (3)	C32—H32C	0.9600
C13—C14	1.361 (3)	C33—O7	1.212 (3)
C13—H13A	0.9300	C33—N1	1.293 (3)
C14—H14A	0.9300	C33—H33A	1.0042
C15—N2	1.302 (3)	N2—N3	1.344 (3)
C15—N5	1.315 (3)	N3—N4	1.279 (3)
C16—C17	1.476 (3)	N4—N5	1.323 (3)
C16—H16A	0.9600	N5—H5A	0.8600
C16—H16B	0.9600	N6—N7	1.334 (3)
C16—H16C	0.9600	N7—N8	1.274 (3)
C17—C20	1.331 (3)	N8—N9	1.333 (3)
C17—N10	1.366 (3)	N9—H9A	0.8600
C18—O1	1.225 (2)	N10—H10B	0.8600
C18—N11	1.317 (3)	N11—H11B	0.8600
C18—N10	1.346 (3)	N12—H12A	0.8600
C19—N11	1.441 (3)	N13—H13B	0.8600
C19—C20	1.504 (3)	O1W—H1WA	0.8499
C19—C24	1.509 (3)	O1W—H1WB	0.8500
C19—H19A	0.9800	C5—H5D	0.9601
C20—C21	1.448 (3)	C5—H5E	0.9601
C21—O5	1.185 (3)	C5—H5B	0.9599
C21—O6	1.330 (3)		
			
C4—C1—C6	121.78 (18)	H22A—C22—H22B	108.0
C4—C1—C2	120.85 (18)	C22—C23—H23A	109.5
C6—C1—C2	117.29 (17)	C22—C23—H23B	109.5
N13—C2—C1	109.72 (16)	H23A—C23—H23B	109.5
N13—C2—C9	109.77 (16)	C22—C23—H23C	109.5
C1—C2—C9	112.83 (16)	H23A—C23—H23C	109.5
N13—C2—H2A	108.1	H23B—C23—H23C	109.5
C1—C2—H2A	108.1	C25—C24—C29	117.6 (2)
C9—C2—H2A	108.1	C25—C24—C19	121.88 (18)
O2—C3—N13	122.93 (19)	C29—C24—C19	120.47 (18)
O2—C3—N12	121.02 (18)	C26—C25—C24	121.3 (2)
N13—C3—N12	116.05 (18)	C26—C25—H25A	119.3
C1—C4—N12	119.62 (18)	C24—C25—H25A	119.3
C1—C4—C5	127.02 (19)	C25—C26—C27	120.3 (2)
N12—C4—C5	113.36 (18)	C25—C26—H26A	119.8
O3—C6—O4	122.1 (2)	C27—C26—H26A	119.8
O3—C6—C1	127.3 (2)	C28—C27—C26	118.5 (2)
O4—C6—C1	110.62 (17)	C28—C27—C30	118.56 (19)
O4—C7—C8	107.5 (2)	C26—C27—C30	122.9 (2)
O4—C7—H7A	110.2	C29—C28—C27	120.9 (2)
C8—C7—H7A	110.2	C29—C28—H28A	119.5
O4—C7—H7B	110.2	C27—C28—H28A	119.5
C8—C7—H7B	110.2	C28—C29—C24	121.3 (2)
H7A—C7—H7B	108.5	C28—C29—H29A	119.4
C7—C8—H8A	109.5	C24—C29—H29A	119.4
C7—C8—H8B	109.5	N6—C30—N9	107.9 (2)
H8A—C8—H8B	109.5	N6—C30—C27	125.0 (2)
C7—C8—H8C	109.5	N9—C30—C27	127.0 (2)
H8A—C8—H8C	109.5	N1—C31—H31A	109.5
H8B—C8—H8C	109.5	N1—C31—H31B	109.5
C14—C9—C10	118.58 (18)	H31A—C31—H31B	109.5
C14—C9—C2	121.16 (18)	N1—C31—H31C	109.5
C10—C9—C2	120.26 (17)	H31A—C31—H31C	109.5
C11—C10—C9	120.90 (18)	H31B—C31—H31C	109.5
C11—C10—H10A	119.5	N1—C32—H32A	109.5
C9—C10—H10A	119.5	N1—C32—H32B	109.5
C10—C11—C12	120.31 (19)	H32A—C32—H32B	109.5
C10—C11—H11A	119.8	N1—C32—H32C	109.5
C12—C11—H11A	119.8	H32A—C32—H32C	109.5
C13—C12—C11	119.11 (19)	H32B—C32—H32C	109.5
C13—C12—C15	121.13 (18)	O7—C33—N1	124.6 (3)
C11—C12—C15	119.75 (18)	O7—C33—H33A	126.2
C14—C13—C12	120.26 (19)	N1—C33—H33A	109.1
C14—C13—H13A	119.9	C33—N1—C31	120.3 (2)
C12—C13—H13A	119.9	C33—N1—C32	122.1 (2)
C13—C14—C9	120.81 (19)	C31—N1—C32	117.6 (2)
C13—C14—H14A	119.6	C15—N2—N3	105.71 (18)
C9—C14—H14A	119.6	N4—N3—N2	110.67 (18)
N2—C15—N5	108.38 (18)	N3—N4—N5	106.21 (18)
N2—C15—C12	126.34 (19)	C15—N5—N4	109.04 (19)
N5—C15—C12	125.27 (19)	C15—N5—H5A	125.5
C17—C16—H16A	109.5	N4—N5—H5A	125.5
C17—C16—H16B	109.5	C30—N6—N7	106.4 (2)
H16A—C16—H16B	109.5	N8—N7—N6	110.6 (2)
C17—C16—H16C	109.5	N7—N8—N9	106.5 (2)
H16A—C16—H16C	109.5	C30—N9—N8	108.6 (2)
H16B—C16—H16C	109.5	C30—N9—H9A	125.7
C20—C17—N10	119.30 (19)	N8—N9—H9A	125.7
C20—C17—C16	127.56 (19)	C18—N10—C17	123.34 (17)
N10—C17—C16	113.14 (18)	C18—N10—H10B	118.3
O1—C18—N11	123.67 (19)	C17—N10—H10B	118.3
O1—C18—N10	120.24 (18)	C18—N11—C19	124.76 (17)
N11—C18—N10	116.09 (18)	C18—N11—H11B	117.6
N11—C19—C20	108.71 (16)	C19—N11—H11B	117.6
N11—C19—C24	111.60 (17)	C3—N12—C4	123.98 (17)
C20—C19—C24	112.44 (17)	C3—N12—H12A	118.0
N11—C19—H19A	108.0	C4—N12—H12A	118.0
C20—C19—H19A	108.0	C3—N13—C2	125.87 (17)
C24—C19—H19A	108.0	C3—N13—H13B	117.1
C17—C20—C21	120.85 (19)	C2—N13—H13B	117.1
C17—C20—C19	119.56 (18)	C6—O4—C7	115.84 (17)
C21—C20—C19	119.60 (18)	C21—O6—C22	115.97 (19)
O5—C21—O6	122.2 (2)	H1WA—O1W—H1WB	109.5
O5—C21—C20	126.7 (2)	C4—C5—H5D	109.5
O6—C21—C20	111.16 (19)	C4—C5—H5E	109.4
O6—C22—C23	111.0 (2)	H5D—C5—H5E	109.5
O6—C22—H22A	109.4	C4—C5—H5B	109.5
C23—C22—H22A	109.4	H5D—C5—H5B	109.5
O6—C22—H22B	109.4	H5E—C5—H5B	109.5
C23—C22—H22B	109.4		

### Hydrogen-bond geometry (Å, º)

**Table:**

*D*—H···*A*	*D*—H	H···*A*	*D*···*A*	*D*—H···*A*
N5—H5*A*···O1*W*	0.86	1.80	2.646 (3)	170
N9—H9*A*···O7	0.86	1.82	2.683 (3)	176
N10—H10*B*···O2	0.86	1.98	2.801 (2)	160
N11—H11*B*···N2^i^	0.86	2.26	3.014 (3)	147
N12—H12*A*···O1	0.86	2.04	2.884 (2)	165
N13—H13*B*···O7^ii^	0.86	2.44	3.176 (3)	144
O1*W*—H1*WA*···N8^ii^	0.85	2.17	2.985 (3)	160
O1*W*—H1*WB*···O1^iii^	0.85	2.01	2.677 (2)	135
C5—H5*D*···O3	0.96	2.07	2.817 (3)	133
C16—H16*A*···O5	0.96	2.03	2.781 (3)	133
C26—H26*A*···O7	0.93	2.59	3.431 (3)	151
C32—H32*A*···N3^iv^	0.96	2.53	3.463 (4)	164
C33—H33*A*···N3^iv^	1.00	2.53	3.506 (3)	164

Symmetry codes: (i) −*x*, −*y*+1, −*z*+1; (ii) −*x*+1, −*y*, −*z*+1; (iii) *x*, *y*, *z*−1; (iv) *x*+1, *y*−1, *z*+1.
